# A Kinesio Taping Method Applied in the Treatment of Postsurgical Knee Swelling after Primary Total Knee Arthroplasty

**DOI:** 10.3390/jcm10132992

**Published:** 2021-07-04

**Authors:** Jaromir Jarecki, Magdalena Sobiech, Karolina Turżańska, Agnieszka Tomczyk-Warunek, Mirosław Jabłoński

**Affiliations:** Chair and Department of Rehabilitation and Orthopaedics, Medical University in Lublin, 20-954 Lublin, Poland; magda.sobiech@op.pl (M.S.); karolina.turzanska@gmail.com (K.T.); a.tomczykwarunek@gmail.com (A.T.-W.); mbjablonski@gmail.com (M.J.)

**Keywords:** Kinesio Taping, osteoarthritis, total knee arthroplasty, ultrasonography

## Abstract

Background The knee is one of the joints in the human body that is most susceptible of osteoarthritis (OA). In the case of advanced-stage OA, total knee arthroplasty (TKA) is a treatment of choice. One modern physiotherapeutic method to support the treatment in the early postsurgical period is Kinesio Taping (KT). The aim of this study is to evaluate the efficacy of KT on swollen subcutaneous tissue after TKA. Materials and methods. The studied group consisted of 23 patients who had received TKA. The mean BMI was 30.60 ± 4.91, and KT was applied between the 3rd and 8th day of the early postoperative period. The control group was constituted by 22 patients who had received TKA. The mean BMI was 30.41 ± 6.00, and KT was not applied. On the 3rd and 8th day after TKA, in all patients, the swelling of the shin, range of motions (ROM), and pain were measured using ultrasound, a goniometer, and a VAS scale, respectively. Results. In the KT group, the lateral measurement at the top of the head of the fibula significantly decreased between the 3rd and 8th day (11.47 ± 0.76 vs. 9.76 ± 0.77; *p* = 0.0004). The knee flexion angle on day 3 was statistically significantly different from that on day 8 (48.61 ± 3.08 vs. 72.74 ± 3.92; *p* = 0.00004). The evaluation results for severity of pain using the VAS scale on day 3 were statistically significantly higher than those on day 8 (5.74 ± 0.25 vs. 4.30 ± 0.25; *p* = 0.00006). In the group of patients to whom KT was not applied, the lateral measurement at the top of the head of the fibula on day 3 was not statistically significantly different from that on day 8 (10.323 ± 0.828 vs. 10.273 ± 0.995; *p* = 0.9227). The knee flexion angle in the group that did not receive KT on day 3 was statistically significantly different from that on day 8 (45.182 ± 3.654 vs. 59.909 ± 4.817; *p* = 0.0006). The severity of pain evaluated using the VAS scale on day 3 was statistically significantly different from that on day 8 (6.227 ± 0.146 vs. 4.864 ± 0.190; *p* = 0.0001). Conclusions. KT is an effective method for improving subcutaneous drainage and decreasing subcutaneous tissue. However, KT does not affect postoperative pain and ROM.

## 1. Introduction

The knee joint is on the list of human joints that are highly prone to degeneration [1,2]. The disease pathophysiology is still poorly understood and is under investigation. It is widely accepted that knee OA is multifactorial in origin [3].

The development of degenerative changes is accompanied by increasing pain, a growing limitation in the range of motion, and swelling. Along with the progression of the condition, the axis of the limbs becomes distorted. In the majority of cases, this is manifested as bowlegs, resulting from damage to the medial compartment of the knee joint.

If no improvement of the knee functions is observed after physiotherapy and pharmacological treatment, while a clear advancement of degenerative changes is visible through an X-ray, the first choice of treatment is primary total knee arthroplasty (TKA) [4]. This surgery aims to reduce pain and improve the motoric function of the joint. The number of knee joint arthroplasty surgical procedures is on the rise all over the world. Arguably, an increase in the population of the elderly, whose joints are more prone to the development of serious degenerative changes, is a strong contributing factor. Simultaneously, more and more, young and middle-aged people, who tend to exercise excessively, are suffering from joint injuries, which cause permanent and irreversible microdamage that can, later on, develop into degenerations and acute pain.

A full recovery after a complex knee joint arthroplasty depends on many factors, including, inter alia, the acceleration of degenerative changes, surgical methods, and periarticular damage [5,6,7]. Physiotherapy is essential for a patient’s recovery plan and motion improvement in the postsurgical period. In the first days after the surgery, one of the main problems that patients encounter is postsurgical pain in the operated limb and subcutaneous tissue swelling, which hinder motion improvement.

One modern physiotherapy method supporting the treatment of the knee joint applied on a daily basis and recommended even at an early stage of the postsurgical period is KT. In 1973, Dr. Kenso Kase presented special techniques involving the application of an elastic tape (KT), which has parameters similar to those of the skin, e.g., weight and thickness, directly to the skin. Depending on the target effect and owing to the use of an adequate technique of application, the tape may, e.g., relieve pain, decrease swelling, provide mechanical corrections, and/or protect the joint while exercising [8,9].

The aim of the study is to evaluate the influence of the application of KT on the reduction of subcutaneous tissue swelling after primary total knee arthroplasty.

At an early stage after TKA, patients frequently develop calf lymphatic oedemas, which decrease ROM, causing pain and resulting in the compression of superficial veins. One of the techniques introduced to improve the status is KT. In this study, to assess its efficacy in reducing subcutaneous tissue, the oedema was measured using ultrasound, which was not applied in previous studies.

The authors hypothesize that the application of KT enhances the reduction of oedema in the calf after TKA, which may improve ROM.

## 2. Material and Methods

The studied group consisted of 55 patients with advanced OA who underwent primary TKA in the Department and Clinic of Rehabilitation and Orthopaedics SPSK-4 Hospital in Lublin in the years 2016–2018. The study design was accepted by the local ethics committee and met the standards set by the Declaration of Helsinki. The approval of the Local Board of Ethical Committee number was KE-0254/288/2016. Table 1 shows the details of the clinical and demographic characteristics of the studied group.

The TKA procedure was performed by one experienced surgeon using a standard technique and the same type of implant (model Genesis II, Smith&Nephew, Cordova, TN, USA). The studied group was randomly divided into two subgroups according to the management procedure applied in the early postsurgical period. The first group consisted of 23 patients, who, in the early postsurgical period, were treated with KT (the method described later) as an additional management tool alongside the standard one. The second group, consisting of 22 patients, was subjected to the standard management procedure, as described below.

On day 1 after the surgery, each patient from both groups, i.e., the treatment one and the clinical control, underwent a standard operating procedure (SOP) of rehabilitation in the hospital. This comprised anti-thrombotic exercises on the lower limbs, isometric exercises on the quadricep, gluteus, and calf muscles, diaphragmatic breathing exercises, active exercises on the free knee joint flexion, and passive–mechanical exercises using a continuous passive motion (CPM) machine. On day 2 after the surgery, the patients were actively lifted to an upright position by means of orthosis. On day 2 or 3, the drainage from the surgical wound was removed. Additionally, on day 1 after the surgery, in both groups, cold compresses, such as Cold Packs, were applied. Each patient was treated pharmacologically with anti-thrombotic drugs, in accordance with the venous thromboembolism prevention recommendations of the Polish Orthopaedic and Traumatology Society, and with painkillers, in accordance with the hospital regulations regarding pain management.

**Figure 1:**
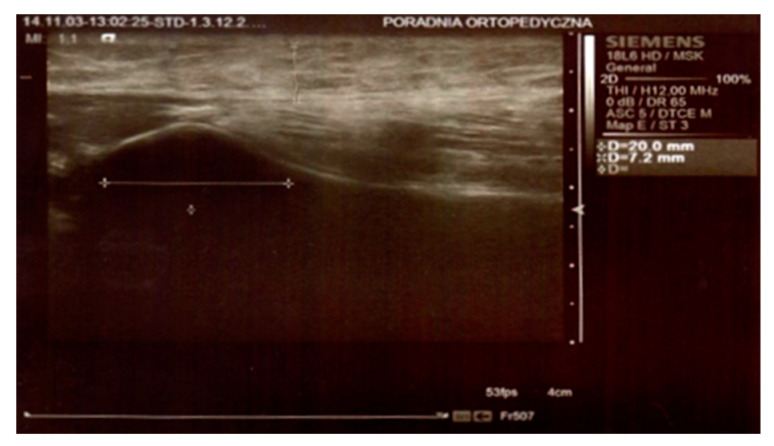
Ultrasound scan. Measurements at the top of the head of the fibula.

**Figure 2:**
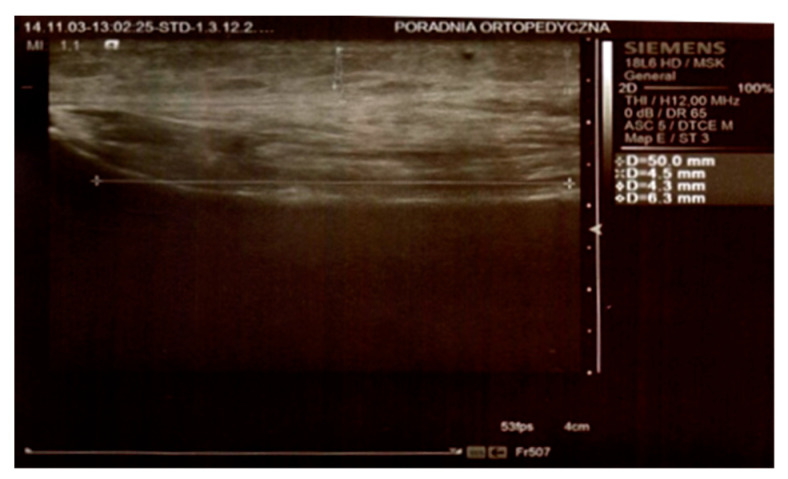
Ultrasound scan. Measurements 25 mm below the neck of the fibula and 50 mm below the neck of the fibula.

In both groups, on day 3 and 8 after the surgery, the thickness of the swelling was determined by an ultrasound scan by means of Simens ACUSON S2000 HELX EVOLUTION with a 18L6 HD linear head. The parameters were measured at the level of the top of the head of the fibula, 25 mm below the neck of the fibula, and 50 mm below the neck of the fibula on the longitudinal axis of the limb (Figure 1 and Figure 2).

**Figure 3:**
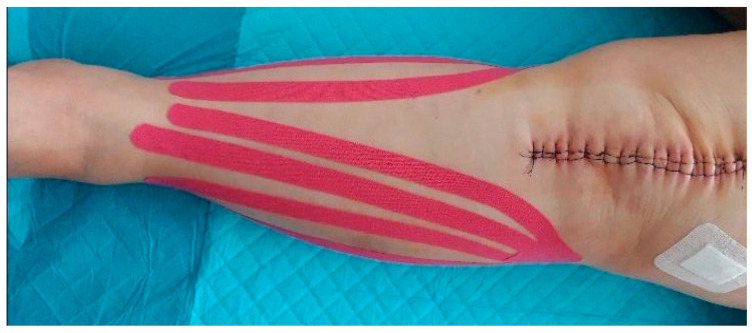
KT application (calf–anterior view).

**Figure 4:**
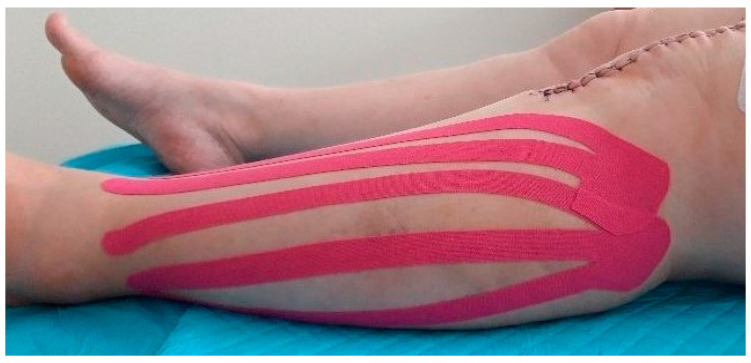
KT application (calf–lateral view).

After the ultrasound scanning on day 3 after the surgery in the first KT group, a 10 mm wide KT (K-Active Tape Classic–Nitto Denko Corporation, 101, Sunada, Shimonome, Iwadeyama, Osaki, Miyagi, 989-6493, Japan) in the shape of a fan was applied to the side of the calf. The proximal points (the base of the tape) were located loosely at the level of the hollow of the knee next to the lymph nodes, the head of the fibula, and on the medial part of the knee. The tapes were located laterally and medially, close to the downward end, according to the surgical approach. The distal points, or tails, were applied with a 15% tape tension and stretched from the lateral malleolus and medial tibia (Figure 3 and Figure 4). After five days (day 8 after the surgery), the tapes were removed, and the swelling was again examined in the manner described above. The initial time of the KT application was chosen on the 3rd day after surgery, because the patients then started walking using a rehabilitation balcony. The choice of the 8th day as the final time was connected with the patients’ discharge. Additionally, after five days, the tapes usually came unstuck and lost their function.

**Figure 5:**
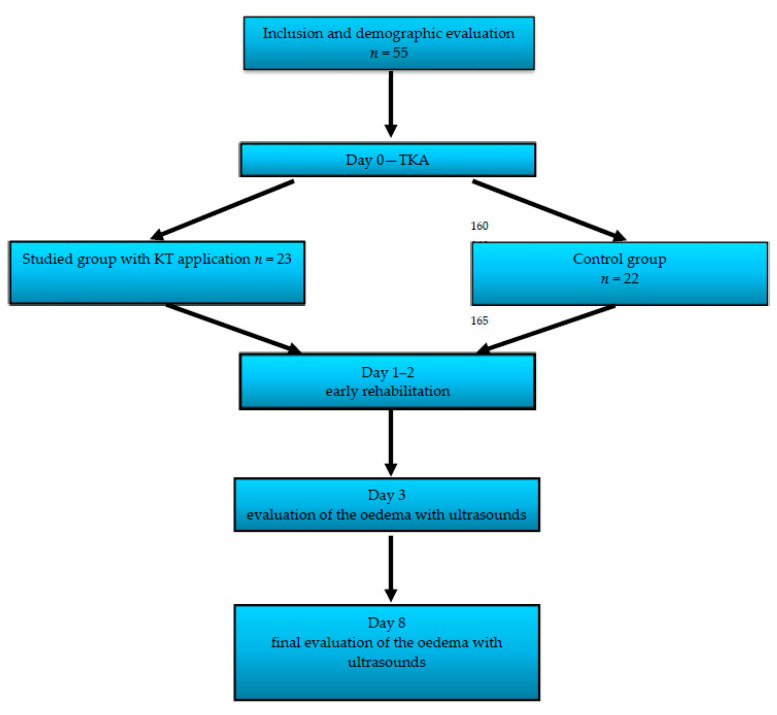
Flowchart of the study design.

In addition to ultrasound measurements, on day 3 and 8 after the surgery, the flexion range of motion of the knee after TKA was examined using the Saehan Rulong 360° goniometer. The severity of pain was evaluated on the VAS numeric scale, ranging from 1 to 10 (Figure 5).

Statistical analyses were conducted with the Polish version of the Statistica 12 software, and *p* < 0.05 was considered significant. The normal distribution was determined by means of the Shapiro-Wilk test. The results before and after the application were compared in pairs using the parametric test t for dependent samples or the nonparametric Wilcoxon’s test. Statistically significant differences were highlighted using various lower-case letters in the superscript (*p* < 0.05).

## 3. Results

KT was tolerated well by all the patients, and neither the daily activities nor the rehabilitation process were limited.

In the KT group, the lateral measurement at the top of the head of the fibula significantly decreased during the analyzed period (3D vs. 8D: 11.7 ± 0.76 vs. 9.76 ± 0.77; *p* = 0.0004). The lateral measurement of 25 mm below the neck of the fibula tended to decrease (3D vs. 8D: 10.15 ± 0.82 vs. 8.99 ± 0.79; *p* = 0.0690). The lateral measurement of 50 mm below the neck of the fibula before (9.54 ± 0.78) and after the application (8.80 ± 0.85) did not differ significantly (*p* = 0.2669). The knee flexion angle significantly increased (3D vs. 8D: 48.61 ± 3.08 vs. 72.74 ± 3.92; *p* = 0.00004). The severity of pain, assessed according to the VAS scale, decreased in time after the surgery (3D vs. 8D: 5.74 ± 0.25 vs. 4.30 ± 0.25; *p* = 0.00006). Table 2 shows the results obtained in detail.

**Figure 6:**
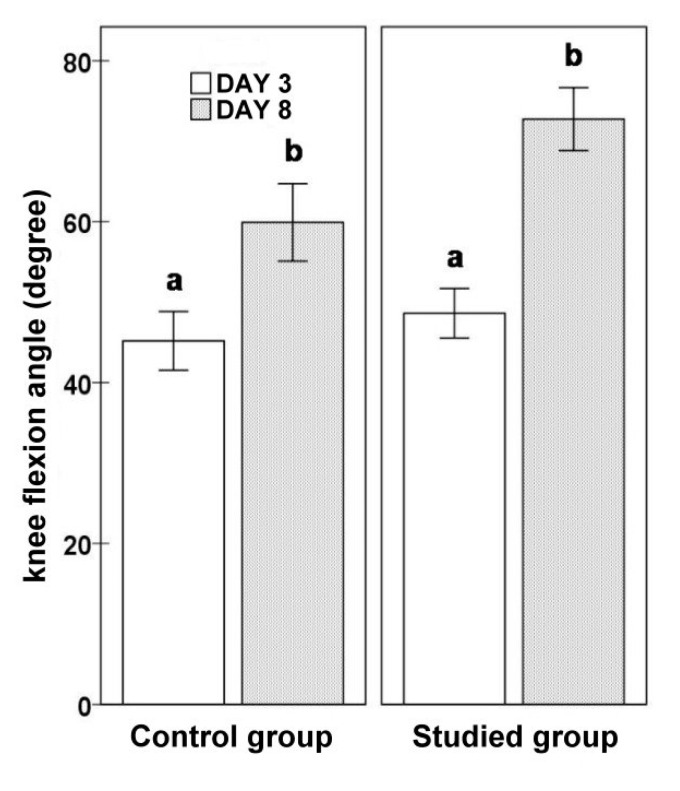
Evaluation of the knee joint flexion angles in the treatment and clinical control groups.

**Figure 7:**
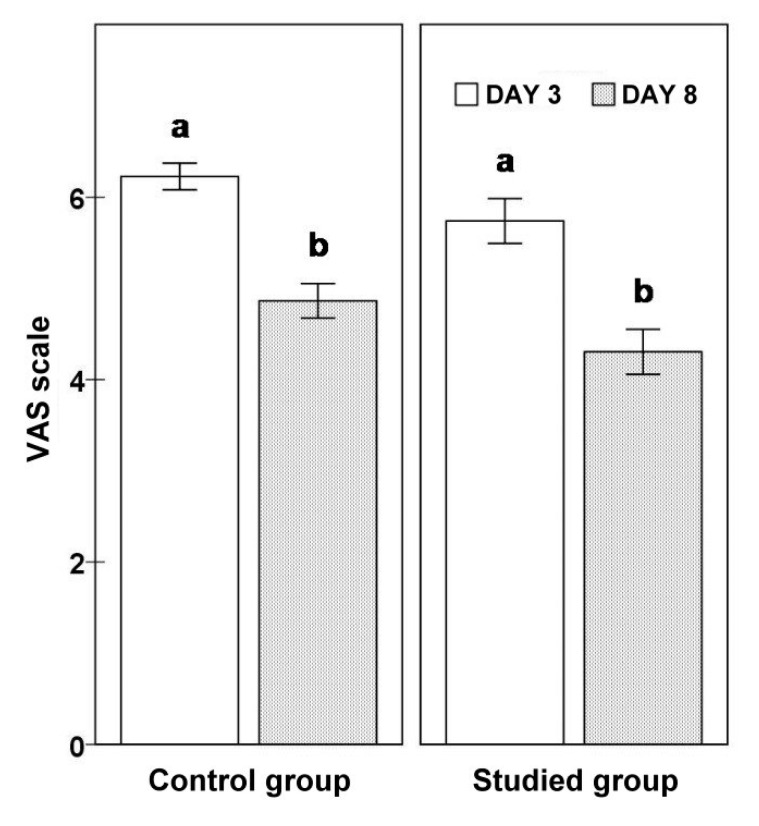
Evaluation of the VAS scale in the treatment group and clinical control groups.

For the group of patients that did not receive KT, the lateral measurements at the top of the head of the fibula on day 3 did not significantly differ from those on day 8 (3D vs. 8D: 10.32 ± 0.83 vs. 10.27 ± 0.99; *p* = 0.9227). The same was confirmed for the lateral measurements of 25 mm below the neck of the fibula (3D vs. 8D: 9.79 ± 0.71 vs. 9.71 ± 0.94; *p* = 0.8757) and the lateral measurements of 50 mm below the neck (3D vs. 8D: 9.84 ± 0.76 vs. 9.22 ± 0.87; *p* = 0.2601). The knee flexion angle in the non-KT group measured on day 3 was significantly higher than that measured on day 8 (45.18 ± 3.65 vs. 59.91 ± 4.82; *p* = 0.0006) (Figure 6). The severity of pain evaluated using the VAS scale significantly decreased during the analyzed period (3D vs. 8D: 6.23 ± 0.15 vs. 4.86 ± 0.19; *p* = 0.0001) (Figure 7). Table 3 shows the results obtained in detail.

When comparing the studied subgroups at the beginning of the measurements, the groups did not significantly differ. There were no significant differences at the end of the study either. However, a statistical trend was reported with respect to the higher values of the knee flexion angle and lower values on the VAS scoring scale in patients who received KT. Table 4 shows the comparative results in detail.

## 4. Discussion

KT, or dynamic taping, involves applying a tape with a weight and thickness similar to the parameters of human skin. KT does not limit motion, and it is waterproof and breathable owing to its wavy texture. KT is based on amplifying the natural processes of the self-healing of the human body. Taping the skin makes the skin lift upwards, which expands the space between the dermis and fascia and thus forms a track for the lymph and blood to flow. Consequently, the lymphostasis and swelling are reduced [8,9,10].

One of the theories that explain the pain-relieving effect is that by lifting the skin, KT reduces the pressure on subcutaneous nociceptors [11,12]. This method is conducive to the normalization of muscle-fascia tension and the correction of the skin-fascia position. Additionally, the proprioception is improved. The reconstruction of proprioception plays a major role in the physiotherapy of joints after surgical procedures [13]. Dynamic taping has an undeniably positive effect on proprioception. The pressure and tension of the tape trigger mechanoreceptors in the skin, which inform about the joint’s position. In a study by T. Halseth, after the application of KT, the proprioception was improved in patients with ankle joint distortion [14]. Additionally, in a study by Kurt et al., the proprioception in patients with a patella-thigh joint conflict was improved in the group where KT was applied to the knee [15].

This method is commonly used for sports injuries of soft tissues, such as shoulder joint injury, back pain, and lymphedema [16,17,18]. Applying it in physiotherapy is a relatively new but thriving concept. Lately, there have been more and more studies seeking to prove its efficacy.

The evaluation the efficacy of KT in relieving pain is the subject of numerous articles. Gonzalez-Iglesias et al. studied the influence of the complex application on pain sensations in patients after a whiplash-type injury. His research proved that in comparison to the clinical control group, the pain sensations were smaller in the KT group by 2 points on a numerical scale of pain [19]. In their studies, Boguszewski, Muray et al. reported a reduction of pain and expansion of the range of motion in the knee joint in patients after an ACL reconstruction with the application of dynamic taping in comparison to a group of patients that did not receive taping [12,20]. E. Kaya Multu et al. also obtained a statistically significant result, which confirms the pain-relieving effect of KT in patients with knee joint degeneration [21]. Our studies showed a reduction of pain according to the VAS scale in both groups, with a slightly more prominent value in the KT group. The research by Laborie et al. showed no differences in the pain evaluation results in patients after ACL [22]. Kachanathu S. J. et al. and Added M. A. et al. assessed the application of KT in chronic back pain in comparison to a group of patients subjected to conventional pain treatment, i.e., manual therapy and exercise, and showed no statistically significant differences [23,24].

The evaluation of the expansion of the range of motion in joints is another issue that has been thoroughly analyzed. Some researchers compared the ranges of motion of the shoulder joint in patients with the application of tapes. In the group where dynamic taping was applied, the shoulder joint range of motion was wider relative to the clinical control group [25,26]. Our analyses show an improvement of the range of motion of the knee joint in both groups with the application and in the clinical control group. When the results of the two groups are juxtaposed, the recorded differences are not statistically significant. Similar results were reported by other researchers while evaluating ROM [21,23,27,28].

KT has a wide application in the treatment of lymphoedema. Numerous studies revealed the anti-swelling effect of taping. In most cases, swelling in patients after oncological surgeries was studied [8,9,20,29,30,31,32]. Boguszewski et al. observed a faster reduction of knee joint swelling in patients who had undergone an ACL reconstruction when KT was applied [20]. Ristov et al. analyzed the facial swelling that occurred on day 1 after a surgical procedure on the temporo-mandibular joint in a group of patients that received lymphatic KT. The authors obtained positive results concerning the reduction of swelling in the KT group [31].

Donec et al. used KT after TKA, which was applied soon after the surgery and showed a pain-relief effect, expanded the range of motion in the joint, and reduced the swelling [33].

Our study is the first to evaluate the use of ultrasound scanning in assessing the subcutaneous tissue swelling in patients treated with dynamic taping. This method is free of biases and allows for precise measurements of the thickness of subcutaneous tissue and a wide range of soft tissue analyses. Moreover, it enables researchers to monitor the records obtained. Non-invasive methods, such as ultrasound scanning, enable physicians to monitor the process of the reduction of subcutaneous tissue swelling on a daily basis.

In summary, the dynamic application of KT is a safe and non-invasive method, which can be used soon after TKA and may improve knee flexion and ease pain in the early postsurgical period.

The study has some possible limitations. First, relatively small groups of patients were included. The groups comprised both genders. However, there is a possibility that the volume and the dynamic of the oedema may differ between females and males. In this study, a single KT application was performed, but it would be interesting to check if multiple KT applications would produce different results. Finally, the oedema was checked only in upper region of the calf. It would also be interesting to evaluate its dynamics in different parts of the lower limb.

## 5. Conclusions

- KT is a safe method that supports the treatment of the knee joint. It can be used on a daily basis and can be applied even in the early postsurgical period.
- The application of KT to reduce subcutaneous tissue swelling after primary TKA accelerates subcutaneous tissue drainage and reduces subcutaneous swelling.
- Using KT does not significantly reduce pain or improve ROM.
- The use of USG scanning is recommended to avoid a biased evaluation of the subcutaneous tissue swelling.

## Figures and Tables

**Table 1:** Demographic and clinical features of the studied group.

Feature	Studied Group	KT Group	Control Group	*p*
Gender M/F	10/35	4/19	6/16	0.3754
Age	66.41 ± 7.36	65.95 ± 7.43	66.9 ± 7.44	0.6856
Body mass index (kg/m^2^)	30.51 ± 5.40	30.60 ± 4.91	30.41 ± 6.00	0.9092

**Table 2:** The results obtained in the subgroups that received Kinesio Taping.

Parameter	Day 3	Day 8	
	Average ± SD	Average ± SD	*p*
At the top of the head of the fibula	11.47 ± 0.76	9.76 ± 0.77	0.0004 *
25 mm below the neck of the fibula	10.15 ± 0.82	8.99 ± 0.79	0.0690
50 mm below the neck of the fibula	9.54 ± 0.78	8.80 ± 0.85	0.2669
Knee flexion angle	48.61 ± 3.08	72.74 ± 3.92	0.00004 *
VAS scale	5.74 ± 0.25	4.30 ± 0.25	0.00006 *

* Statistically significant.

**Table 3:** The results obtained in the subgroups that did not receive Kinesio Taping.

Parameter	Day 3	Day 8	
	Average ± SD	Average ± SD	*p*
At the level of the top of the fibula	10.32 ± 0.83	10.27 ± 0.99	0.9227
25 mm below the neck of the fibula	9.79 ± 0.71	9.71 ± 0.94	0.8757
50 mm below the neck of the fibula	9.84 ± 0.76	9.22 ± 0.87	0.2601
Knee flexion angle	45.18 ± 3.65	59.91 ± 4.82	0.0006 *
VAS scale	6.23 ± 0.15	4.86 ± 0.19	0.0001 *

* Statistically significant.

**Table 4:** Comparisons of the studied subgroups.

Parameters	Kinesio Taping Applied	Kinesio Taping Not Applied	
	Average ± SD	Average ± SD	*p*
**Day 3**			
At the level of the top of the fibula	11.47 ± 0.76	10.32 ± 0.83	0.312
25 mm below the neck of the fibula	10.15 ± 0.82	9.79 ± 0.71	0.7449
50 mm below the neck of the fibula	9.54 ± 0.78	9.84 ± 0.76	0.7801
Knee flexion angle	48.61 ± 3.08	45.18 ± 3.65	0.4537
VAS scale	5.74 ± 0.24	6.23 ± 0.15	0.1339
**Day 8**			
At the level of the top of the fibula	9.76 ± 0.77	10.27 ± 0.99	0.6842
25 mm below the neck of the fibula	8.99 ± 0.79	9.71 ± 0.94	0.5553
50 mm below the neck of the fibula	8.80 ± 0.85	9.22 ± 0.87	0.7350
Knee flexion angle	72.74 ± 3.92	59.91 ± 4.82	0.1069
VAS scale	4.30 ± 0.25	4.86 ± 0.19	0.1119

## Data Availability

The data are available from corresponding author on demand.

## Abbreviations

TKA	Total knee arthroplasty
KT	Kinesio Taping
ROM	Range of motion
USG	Ultrasonography
